# Indicators and Criteria of Consciousness in Animals and Intelligent Machines: An Inside-Out Approach

**DOI:** 10.3389/fnsys.2019.00025

**Published:** 2019-07-16

**Authors:** Cyriel M. A. Pennartz, Michele Farisco, Kathinka Evers

**Affiliations:** ^1^Department of Cognitive and Systems Neuroscience, Swammerdam Institute for Life Sciences, University of Amsterdam, Amsterdam, Netherlands; ^2^Research Priority Area, Brain and Cognition, University of Amsterdam, Amsterdam, Netherlands; ^3^Centre for Research Ethics and Bioethics, Uppsala University, Uppsala, Sweden; ^4^Biogem, Biology and Molecular Genetics Institute, Ariano Irpino, Italy

**Keywords:** awareness, bird, episodic memory, goal-directed behavior, illusion, robot, rodent, visuospatial behavior

## Abstract

In today’s society, it becomes increasingly important to assess which non-human and non-verbal beings possess consciousness. This review article aims to delineate criteria for consciousness especially in animals, while also taking into account intelligent artifacts. First, we circumscribe what we mean with “consciousness” and describe key features of subjective experience: qualitative richness, situatedness, intentionality and interpretation, integration and the combination of dynamic and stabilizing properties. We argue that consciousness has a biological function, which is to present the subject with a multimodal, situational survey of the surrounding world and body, subserving complex decision-making and goal-directed behavior. This survey reflects the brain’s capacity for internal modeling of external events underlying changes in sensory state. Next, we follow an inside-out approach: how can the features of conscious experience, correlating to mechanisms inside the brain, be logically coupled to externally observable (“outside”) properties? Instead of proposing criteria that would each define a “hard” threshold for consciousness, we outline six indicators: (i) goal-directed behavior and model-based learning; (ii) anatomic and physiological substrates for generating integrative multimodal representations; (iii) psychometrics and meta-cognition; (iv) episodic memory; (v) susceptibility to illusions and multistable perception; and (vi) specific visuospatial behaviors. Rather than emphasizing a particular indicator as being decisive, we propose that the consistency amongst these indicators can serve to assess consciousness in particular species. The integration of scores on the various indicators yields an overall, graded criterion for consciousness, somewhat comparable to the Glasgow Coma Scale for unresponsive patients. When considering theoretically derived measures of consciousness, it is argued that their validity should not be assessed on the basis of a single quantifiable measure, but requires cross-examination across multiple pieces of evidence, including the indicators proposed here. Current intelligent machines, including deep learning neural networks (DLNNs) and agile robots, are not indicated to be conscious yet. Instead of assessing machine consciousness by a brief Turing-type of test, evidence for it may gradually accumulate when we study machines ethologically and across time, considering multiple behaviors that require flexibility, improvisation, spontaneous problem-solving and the situational conspectus typically associated with conscious experience.

## Introduction

Over the past decades, it has become increasingly important to assess consciousness in non-human and/or non-verbal beings, such as animals and—perhaps in a not too distant future—machines accomplishing sophisticated cognitive tasks. Examples that underscore the relevance of such assessments include the question of animal sentience (including a capacity to suffer pain) in relation to animal welfare in bio-industrial farming, in procedures for ritual slaughter, animal experimentation for biomedical purposes, but also in domestic pet keeping. Second, in the spectrum of Artificial Intelligence (AI) techniques, we find computers and robots performing complex tasks, such as playing Jeopardy! and other knowledge games, Alpha-Go or navigating through indoor as well as outdoor environments while negotiating irregular terrains (Murphy et al., 2011; Ferrucci et al., 2013; Hassabis et al., 2017). AI is currently developing at such a rapid pace that supra-human performance can be claimed for several games, acquired through self-play (Silver et al., 2018). This development gives rise to the question whether machines may have a level of sentience or consciousness, and ipso facto may be entitled to certain rights and moral status. A third domain where assessment of consciousness based on non-verbal criteria is highly relevant is represented by people who cannot linguistically express themselves, ranging from infants to patients suffering from global aphasia, deeply demented patients, patients in a minimally conscious state or locked-in syndrome.

In this review article, we will suggest operational criteria that can facilitate researchers and policymakers in a range of connected fields of study (neuroscience, AI, psychology and behavioral sciences, medicine, philosophy, ethics and animal welfare) in attributing levels of consciousness to non-verbal beings which may assume varying states of information processing (such as sleep and wakefulness). Such criteria aid us in determining who or what may be conscious, but we must note that an entity might be conscious even if it fails to satisfy them. Like in the neuroimaging assessment of residual consciousness in patients with disorders of consciousness (Schnakers et al., 2009; Owen, 2014), we should keep in mind here that absence of evidence is not the same as evidence of absence of consciousness. At the same time, one must be cautious not to attribute consciousness to any arbitrary object in nature without further argumentation. As we will argue, many criteria can be best circumscribed as “indicators of consciousness,” a term which avoids the connotation of “criterion” with a hard threshold in judging or deciding whether someone or something is conscious or not.

With “operational” we mean that such indicators of consciousness should be sufficiently concrete to apply them in the practice of observing the behavior or cognitive performance of the subject under scrutiny. We will use the term “consciousness” interchangeably with “awareness,” without denying that a distinction between them has been drawn in some previously proposed frameworks (Farisco et al., 2017; Kastrup, 2017). We will depart from a brief overview of features that may be reasonably attributed to conscious experience, contextualize these properties in terms of biological function, and then proceed to translate such properties into observable behaviors, physiological responses, anatomical traits and/or internally generated phenomena (such as computational operations reaching a certain degree of complexity) that may be considered indicators of consciousness. That this approach is necessarily indirect and does not yield definitive proof, but rather indications for consciousness, is an unavoidable consequence of the epistemological position that the conscious experience of other beings, including other humans, is essentially subjective and that relevant knowledge about another subject’s experience is always inferential (Evers and Sigman, 2013).

Defining such indicators of consciousness is not an easy task and we refer to previous work in this direction (Weiskrantz, 1995; Griffin and Speck, 2004; Seth et al., 2005; Butler and Cotterill, 2006; Edelman and Seth, 2009; Pennartz, 2015). First and foremost, each definition of a criterion or indicator of consciousness depends on assumptions being made on what a conscious experience is, if and how it normally arises from a brain-body complex interacting with its environment, what its biological function may be, and how it relates to basic computational operations performed by the brain or artificial machinery aiming to replace it, either fully or in part. In this respect, the current article differs from previous outlines of consciousness criteria that were based, in part, on Integration Information Theory (IIT; Boly et al., 2013; Tononi et al., 2016), Global Neuronal Workspace (Dehaene and Changeux, 2011; Baars et al., 2013), or combinations thereof (Seth et al., 2005). As explained in more detail below, we start from the view that consciousness has a biological function: it presents the subject with a multimodal, situational survey of its surrounding world (including its own body), which subserves the kind of complex decision-making that is associated with goal-directed, deliberate, planned behavior (Pennartz, 2015, 2018). This survey takes the form of a dynamic “world model” or representation that is coded or given a symbolic expression by groups of interconnected thalamo-cortical areas, which are essentially making a “best guess” of the external causes underlying changes in sensory inputs reaching the brain (see Gregory, 1980; Mumford, 1992; Friston, 2010; Olcese et al., 2018b). In line with this, a previously proposed framework, departing from philosophy (i.e., the Intrinsic Consciousness Theory; Farisco et al., 2017), similarly advocates a view of consciousness as a modeling and goal-directed activity, reflecting the brain’s ability to model the world and body, allowing the subject to infer the surrounding world’s state on the basis of such models. In Intrinsic Consciousness Theory, consciousness is an abductive, probabilistic and dynamic feature of the brain that is shaped by interaction with the world and largely depends on previous experience. This abductive feature is realized through the brain’s ability to infer the best explanation (or model) of the data received from its surroundings *via* sensory receptors. Although originally developed in the context of studies on the brain, Intrinsic Consciousness Theory does not exclude in principle that the functions we associate with consciousness may be manifested by artificial subjects.

While it has not been uncommon in the past to associate consciousness with volition and voluntary decision-making (Crick and Koch, 2003), these latter two concepts have been difficult to test or quantify in practice, whereas goal-directed behavior (see below) is a well-defined concept that has been operationalized in studies on animal behavior (Balleine and Dickinson, 1998; Dickinson, 2012). However, it has remained unclear whether goal-directed behavior may deliver equally clear indicators of consciousness for assessing conscious processing in intelligent machines, and to what extent the coupling of goal-directed behavior to consciousness is unconditionally valid or conditional on further requirements. As far as evidence for goal-directed behavior may be lacking in certain animal species, the question arises whether other indicators of consciousness may be derived from the concept of conscious experience as a multimodal, situational survey.

**Figure 1:**
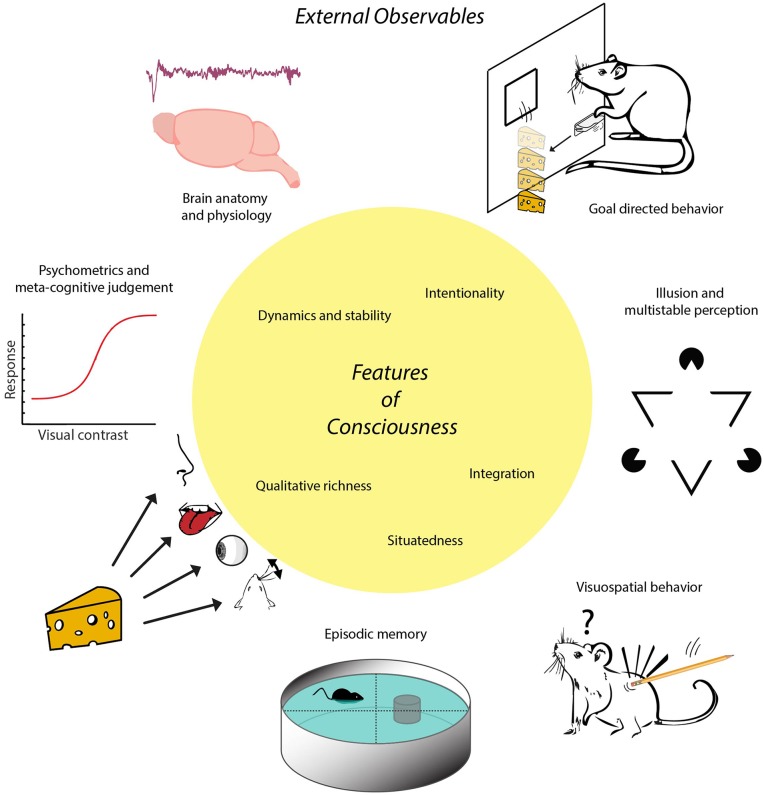
Schematic of the proposed inside-out approach. Conscious experience is conceptualized as a multimodal situational survey having certain characteristic features, represented here in the central yellow circle. These features imply that animals or other subjects having consciousness will exhibit certain externally observable characteristics that can be established through e.g., anatomical, physiological or behavioral studies. For instance, possessing the feature of situatedness entails a more sophisticated set of visuospatial behaviors than displayed by subjects whose behavior is governed by (non-consciously processed) singular stimulus-response relationships. Having this feature in combination with memory capacities enables the formation of episodic memory, defined *via* the conscious recall of occurrences specified by event identity, place and time. Note, first, that there is no one-to-one mapping between features of consciousness and external observables in this scheme; a given feature may be coupled to multiple external observables and vice versa. Second, external observables are rendered here by icons symbolizing more complex processes. For instance, goal-directed behavior is symbolized here by instrumental learning to acquire cheese reward by lever pressing, whereas it also requires sensitivity to devaluation and degradation of action-outcome contingency. The trace placed above “Brain anatomy and physiology” denotes an EEG fragment from non-REM (non-Rapid Eye Movement) sleep. The term “inside-out approach” does not entail that the contents of experience are internally situated (i.e., within the brain) but refers to the localization of neural mechanisms underlying experience inside the brain.

In short, we will consecutively address five questions: (i) What do we mean with “consciousness,” and which set of features can we uniquely associate with subjective experience? (ii) How many distinctions between goal-directed behavior and habitual actions help delineate indicators of consciousness in non-human and/or non-verbal subjects? (iii) Which other indicators of consciousness may be used in addition to those based on goal-directedness? (iv) To what extent can theoretically derived, quantitative measures of brain activity be used as indicators of consciousness? and (v) Can the indicators of consciousness held to be applicable to non-human animals be extrapolated to apply to intelligent machines as well—and if not, which operational approach may be proposed in addition or instead? In addressing these questions, we will follow an “inside-out” approach: starting from features that can be attributed to subjective experience, correlating to neural mechanisms inside the brain, we continue with outlining a biological function of consciousness in relation to behavior, and hence infer externally observable (“outside”) properties that can be reasonably associated with consciousness in terms of brain anatomy, physiology and behavior (Figure 1). Thus, the approach is to study how subjects “act out” their multimodal situational survey through externally observable phenomena. This approach is pluralistic in the sense that it is open to recognizing the validity of multiple sources of potential evidence for consciousness (e.g., physiological, behavioral, anatomical).

## What Does “Consciousness” Mean and What Are Its Key Properties?

In clinical practice, states of consciousness are mainly probed by prompting patients to report events with accuracy, usually by verbal expression (Laureys et al., 2004; Seth et al., 2005; Monti et al., 2010; Bruno et al., 2011). For instance, a physician may ask a patient whether he or she feels a touch to the skin, hears a particular tone or sees a stimulus presented on a screen. Such perceptual testing is routinely supplemented with verbal commands to generate a voluntary response; the physician may ask the patient: “can you raise your left arm” or “can you tell me your name” (Monti et al., 2009, 2010). Despite their practical usefulness, these traditional bedside criteria suffer from the limitation that they depend on the motor capacities of the individual, whereas patients suffering from disorders of consciousness and lacking capacities for behavioral expression may have residual consciousness that these traditional criteria perforce fail to detect (Schnakers et al., 2009; Farisco et al., 2015; Farisco and Evers, 2016). Other patient groups e.g., suffering from paralysis, global aphasia or locked-in syndrome also lack capacities for behavioral communication, yet are recognized to remain conscious (Laureys et al., 2004; Casali et al., 2013; Owen, 2014).

A special case is constituted by patients who are fully locked-in and thus lack any communication channel with their families or medical personnel. Sometimes evidence on their conscious state emerges when they recover and recall from memory what they experienced during their locked-in state (Schiff, 2010; Sitt et al., 2014; Engemann et al., 2018), but in many other cases such *post hoc* evidence for conscious processing cannot be obtained. Evidence for active cognitive processing in behaviorally non-communicative patients may be obtained by functional magnetic resonance imaging (fMRI) imaging in combination with verbal commands to generate differentiated imagery (e.g., spatial vs. motor imagery), but such evidence is not fully conclusive as the findings are compatible with the possibility that ongoing cognitive processing might occur in the absence of awareness (Owen, 2013).

These and other considerations (Pennartz, 2015, 2018) emphasize the need to maintain a dissociation between consciousness *per se* and the repertoire of motor (including verbal) actions humans and non-human subjects may use to express what they experience. Thus, we define consciousness in the first instance by its experiential, sensory nature (Jackendoff, 1987). We take “experience” to denote the complex of conscious sensations occurring during states of wakeful processing of sensory inputs of external origin (perception), imagery (sensations due to internally driven processing under cognitive control) or dreaming (sensations due to internally driven processing, prevalent during REM sleep and less subject to cognitive control; Hobson, 2009). This points to a limitation in the scope of the current article; we do not theoretically deny the possibility of adopting a more inclusive view on subjective experience (see Farisco et al., 2017; Kastrup, 2017). Notably, many neuroscientists use the term “experience” more broadly, referring to life events that happen to organisms in general and lead to functional changes such as learning or memory storage (captured by terms such as “experience-dependent plasticity”; Pennartz, 2018). Perception, imagery and dreaming can be considered to constitute the main forms or modes by which consciousness is subjectively manifested, contrasting to unaware states such as dreamless sleep, anesthesia or coma.

Defining consciousness directly is notoriously difficult because one is forced to resort to related concepts such as wakefulness, experience, feeling and perceiving (or their unaware counterparts, such as deep sleep). When attempting to define these terms in turn, one is forced to resort to circularity. Therefore, we (Pennartz, 2015) and others have previously characterized (healthy, full-blown) conscious experience not by trying to define it directly, but as having a number of key features that characterize consciousness (though not necessarily uniquely; Hassin, 2013; Table 1):

(i) **Qualitative richness**: conscious contents we experience are specified by a wide, varied palette of sensory modalities (vision, audition, somatosensation, olfaction, taste, vestibular sense) and submodalities (e.g., for vision: texture, motion, color, size, shape, depth). This notion does not imply that any experience is required to involve all or most modalities at the same time, but does imply that contents are constituted by modally specific elements that are experienced as distinct from one another (see Pennartz, 2009).
(ii) **Situatedness**: whatever a subject in a healthy, normal condition experiences is set in a specific spatiotemporal situation, i.e., the subject is immersed in a multimodal situation characterized by a specific body position occupying a place in an environmental space and within a temporal framework. With “immersion” we mean that the subject is not looking at its own situation from a distance, but experiences its own body as being within the situation.
(iii) **Intentionality**: experiences are fundamentally about something other than is entailed by the neuronal substrates (“vehicles”) underlying their generation. We can be conscious of exploding fireworks while the brain mechanisms at work to generate this experience are not exploding themselves (Searle, 2004; Olcese et al., 2018b). Our experiences fundamentally depend on an interpretation of the external or internally generated inputs to the brain which are processed by neural mechanisms that are of a different nature than the subjectively experienced contents. This process can alternatively be described as making subjective inferences on the causes of sensory inputs reaching our brain, e.g., on objects emitting light, sound waves or other changes in energy impinging on sensory receptors. This is not to say that the brain would be insensitive to the causes and their ensuing sensory inputs. On the contrary, the latter affect the brain *via* the cranial nerves and spinal cord.
(iv) **Integration**: the elements of a scene or situation we perceive are experienced as a unified whole, and this “in-one-piece” property is not compatible with subjects sustaining different aware experiences at the same time (see Tononi, 2004). We consider integration to be a broad, overarching term that comprises different forms and computational mechanisms, such as binocular fusion (i.e., the merging of different visual information originating from images projected on the left and right eye), temporal integration of visual information across saccades and eye blinks, integration of contours and other Gestalt features into whole objects, and multimodal integration (e.g., perceived audiovisual simultaneity in the presence of physical light-sound delays).
(v) **Dynamics and stability**: brain systems for conscious processing allow for both dynamic changes in experience as well as for short-term stabilization of percepts, e.g., when viewing ambiguous pictures (e.g., Necker cube inversion; Borsellino et al., 1982), binocular rivalry (Tong et al., 1998), experiencing illusions (e.g., a Kanizsa triangle; Seghier and Vuilleumier, 2006; von der Heydt, 2013) or change detection (Huettel et al., 2001). Moreover, when moving one’s eyes and navigating through a stable environment, the subject experiences objects and scene elements as being stably positioned, indicating that the involved brain systems generate stable percepts despite a plethora of dynamic changes in sensory inputs.

**Table 1:** Key features of consciousness.

Qualitative richness	Conscious experience is specified by a wide, varied palette of sensory modalities and submodalities
Situatedness	Conscious experience is set in a specific spatiotemporal situation in which the subject is immersed
Intentionality	Conscious experience is about something other than is entailed by the underlying neuronal substrates; it depends on the subjective interpretation of external or internal inputs to the brain
Integration	The elements of a scene or situation we perceive are experienced as a unified whole
Dynamics and stability	Brain systems for conscious processing allow for both dynamic changes in experience as well as short-term stabilization of percepts

Features (ii) and (iv) are often associated with having a first-person, egocentric perspective (Searle, 1992, 2015) or an “observing self” (Baars et al., 2003). Indeed a subject samples world and body states through one overall sensory apparatus, resulting in a visual perspective that is integrated with body position in space (Pennartz, 2015, 2018), but we add that this notion of egocentric perspective leans rather heavily on the dominance of vision in conscious experience, and is less obvious when subjects consciously manipulate objects or navigate allocentrically through their environments (i.e., experience can be partially object-centered and influenced by allocentric knowledge; Pennartz, 2015).

According to particular schools of thought, further features of consciousness may be added, such as aspects of language (Carruthers, 1989), self-consciousness and “higher-order thought” (HOT; see Seth et al., 2005). HOT theories propose that consciousness critically depends on a form of meta-cognition by which subjects reflect on, evaluate and judge sensory contents (Cleeremans et al., 2007; Lau and Rosenthal, 2011). However, we and others (Koch et al., 2016) have argued elsewhere that human subjects retain the primary, sensory consciousness that we referred to above when such meta-cognitive or reflexive capacities are compromised or lost, for instance because of massive frontal lesions, drug-induced alterations in, or loss of, confidence judgments, or mental illness (Pennartz, 2015; Boly et al., 2017; Storm et al., 2017). Moreover, conscious sensations can exist without being granted a particular ontological status (e.g., as “veridical” or “hallucinatory”) by a separate meta-cognitive system.

Thus, we argue to keep the list of key properties of conscious experience compact, summarizing it as having a multimodal, unified and situational survey of the surrounding world and oneself. This survey is fundamentally inferential and thus subjective, given that more than one interpretation is possible when multiple options for making inferences are available. The multimodal, situational survey offers a “best guess” representation (see Gregory, 1980; Marcel, 1983) of what is happening around us, correcting for rapid sensorimotor changes that would disrupt a stable world view (e.g., eye blinks, saccades) and yet allowing for a dynamic updating. This characterization, however, is very much based on human subjective experience and its occurrence in animals or machines—in this form or in related manifestations—can of course not be directly verified. The core features do not reveal indicators of consciousness by which states of consciousness in non-verbal beings can be directly identified. Nonetheless, we will argue that they do point the way to finding reasonable, although indirect, indicators of consciousness that can be used in practice.

## Biological Function of Consciousness

If one accepts the premise that conscious experience essentially corresponds to having a multimodal, inferential and situational world survey, what function could it subserve? Whereas it is not self-evident that consciousness *does* have a biological function, it is of note that evolution has led to the development of brain systems which are strongly associated with conscious sensing (i.e., thalamo-cortical systems for all modalities except, arguably, olfaction which may not require thalamic functioning; Pennartz, 2015) and pose high demands in terms of energy consumption. This evolutionary development underwrites that consciousness has survival and reproductive value, contributing to a subject’s innate inclination to persist and enhance in its integral functioning (Evers, 2009). To this one may raise the reductionist objection that consciousness could be an epiphenomenon, and that the “real work” for realizing meaningful sensorimotor behaviors is being performed by the neurons, without having to resort to an elusive phenomenon such as consciousness (Churchland, 1984).

However, this counterargument does not hold because of the *functional nature* of conscious experience as characterized above. When the neurons would, hypothetically, execute the “real work” without generating a multimodal, situational survey, consciousness would be lacking and organisms would have to grope around “in the dark” in their daily struggle for survival and reproduction (notably, darkness is only a metaphor in this case because a wholly nonconscious subject would not even be aware of darkness). At first glance, a proponent of the epiphenomenal argument may sit comfortably with this notion because, after all, subjects may seem to survive perfectly well as long as sensory stimuli are properly handled by sensorimotor neural circuits producing effective motor actions. However, the problem with this reasoning is that it only works well for behaviors generated in response to simple stimulus-response situations, such as reflexes and habits. Reflexes ensure simple and very fast motor reactions in response to low-dimensional sensory inputs, such as the heat of a candle flame resulting in immediate withdrawal of a nearby hand. Habits result from overtraining of stimulus-response associations (Balleine and Dickinson, 1998; Dickinson, 2012; de Wit et al., 2018) and thereby come to be executed more automatically and without (or at least with less) consciousness. We may be aware of the global sensory consequences of habitual actions but remain largely unaware of the sensory details or their underlying fine movements (Lisman and Sternberg, 2013; Pennartz, 2018).

Contrasting with reflexes and habits, complex decision-making cannot purely rely on low-dimensional or single-stimulus information to select actions quickly. With “complex” we mean that many variables need to be simultaneously taken into account to reach an optimal decision: not only estimates of single stimuli and their motivational value (in terms of reward or punishment), but also estimates of energetic effort, knowledge about the sensory-specific, social and reproductive consequences of stimuli, and the arrangement of, and coherence between, these variables in space and time. Even objects without explicit motivational value need to be taken into account, for instance, because they may become obstacles if the agent chooses a route to a more distant goal, or because they become instrumental if a different goal is selected in the course of action. Complex decision-making in novel or familiar situations not only requires organisms to have an internal evaluative system, specifying what they need to satisfy homeostatic variables both in terms of reward (acquisition of desirable items such as food and water) and punishment (avoidance or harmful and noxious stimuli), but also to have a model that is optimally informative about their current and future sensory world. Below we will go more deeply into two concepts strongly related to complex decision-making: goal-directed behavior and model-based learning.

It would be extremely difficult to negotiate such complex problem sets if subjects would have to work without some sort of situational survey offering a quick grasp on the situation at hand. We suggest that evolution has solved this problem by developing specialized brain systems actively generating rapidly updatable world surveys. We do not mean to equate consciousness with complex decision-making (or related meta-cognitive processes such as confidence judgments) but claim that consciousness *subserves* (i.e., promotes and supports) it (see Griffin and Speck, 2004). In other words, consciousness is relevant for complex decision-making (which is not to say the former is a necessary condition for the latter). Conscious experience also continues in the absence of overt decisions or motor actions, such as during dreaming, daydreaming or passive observation without motor intentions or planning (Treserras et al., 2009; Pennartz, 2018).

## Indicators of Consciousness in Animals

Departing from the premise that consciousness is functionally linked to complex decision-making, we can now begin to outline indicators of consciousness in animals in the context of their behavior, neuroanatomy and physiology (Figure 1), noting that for several classes of animals, multiple indicators of consciousness will be proposed as contributing evidence in favor or against the presence of consciousness (Table 2).

**Table 2:** Indicators of Consciousness.

Goal Directed Behavior and model-based learning	Goal-directed behavior is driven by a representation of the expected consequences of action and depends on knowledge of actions being causal for obtaining a desirable outcome Model-based learning is defined by the subject building an explicit, internal model of its state space, including specific stimulus–outcome relationships and enabling prospective cognition
Brain anatomy and physiology	In primates, conscious experience is associated with thalamocortical systems. In other vertebrates, similar brain structures indicate the presence of consciousness. In vertebrates, functional analogs of cortex and thalamus may support consciousness
Psychometrics and meta-cognitive judgment	Psychometric properties of stimulus detection and discrimination, coupled to meta-cognitive judgments on perceived stimuli, indicate perceptual similarities between humans and some animal species (e.g., monkeys, rodents, and birds)
Episodic memory	Autobiographical memory, i.e., memory of events (“what”) a subject experienced at a particular place (“where”) and time (“when”), indicates the presence of consciousness because it is linked to it by the definition of declarative memory
Illusion and multistable perception	Susceptibility to illusions and perceptual ambiguity has been demonstrated in non-human primates and several other species, and is coupled to intentionality, the key feature by which conscious systems can interpret sensory information in different ways
Visuospatial behavior	Having a stable, situational survey in the face of ongoing body and eye movements is coupled to specific visuospatial abilities, such as reaching into non-foveated parts of space and identifying objects as being stably positioned

### Goal-Directed Behavior and Model-Based Learning

The distinction between habits and goal-directed behavior has been operationalized by Balleine and Dickinson (1998), Dickinson (2012) and is important for the current debate, even though goal-directed behavior should not be equated with complex decision-making in general. Agents display goal-directed behavior when this behavior is driven by a representation of the expected consequences of action and depends on knowledge of actions being causal for obtaining a desirable outcome (Dickinson, 2012). Operationally, this definition can be tested, first, by studying the consequences of devaluation of the outcome (e.g., by prior satiation of the animal to a particular food) in a behavioral setting where animals learn to react to stimuli by performing actions. Here in goal-directed behavior is marked by a rapid decline in action execution after outcome devaluation, whereas habitual responses to stimuli will persist given the long-term strength of the underlying stimulus-response association (Balleine and Dickinson, 1998). The neural system mediating goal-directed behavior in mammals is thought to comprise a different set of brain structures (i.e., medial prefrontal and orbitofrontal cortices, hippocampus, ventral and dorsomedial striatum) than that implied in habit learning and performance (i.e., sensorimotor cortices and dorsolateral striatum; Balleine and Dickinson, 1998; Corbit and Balleine, 2000; Yin et al., 2004; Pezzulo et al., 2014; O’Hare et al., 2016; Pennartz, 2018); the current evidence on goal-directed behavior as defined above mainly pertains to rodents, marmosets and humans. Even though this definition of goal-directed behavior does not entail that it requires situations and decisions to be “complex” (in the sense meant above) for it to occur, it is reasonable to link the concepts of goal-directed behavior and complex decision-making on two grounds.

First, it allows behaviors to be distinguished from habitual (and by extension reflexive) behaviors, which can be placed largely in the domain of unaware information processing and non-complex decision-making. Second, the set of goal-directed behavior-related brain structures exhibits a strong overlap with brain areas implied in model-based learning, which intimately relates to complex decision-making and thereby contrasts with its habitual counterpart, model-free learning (Daw et al., 2005). Model-based learning is defined by the subject building an explicit, internal model of its state space, including specific stimulus–outcome relationships and enabling prospective cognition and on-the-fly decision-making (Daw and Dayan, 2014; Pennartz, 2018). Sometimes it is held to be identical to learning goal-directed behavior, but it is of note the two concepts are defined differently. Whereas goal-directed behavior emphasizes knowledge of outcome value and the causal dependence of outcome on operant action, model-based learning focuses on the agent building a sensory-specific model of stimulus-action-outcome relationships, on prospective activity that anticipates future events, and the ability to make decisions on the fly, spontaneously, based on generalizable internal models (Daw and Dayan, 2004). The importance of spontaneously arising behavior will be highlighted in “Consciousness in Intelligent Machines and Its Relations to Animal Cognition” section. Despite these different emphases, the two concepts conspire to suggest a common brain system supporting complex, non-habitual learning and planned decision-making, which not only depends on declarative memory recall but also on the on-line situational survey typically associated with consciousness. Recent studies on neurophysiological correlates of goal representations and deliberation in mammals have underscored the common involvement of the set of brain structures already indicated (medial prefrontal and orbitofrontal cortices, hippocampus, ventral and dorsomedial striatum) in both goal-directed behavior and model-based learning (Johnson and Redish, 2005; Hok et al., 2012; Pezzulo et al., 2014; Genovesio et al., 2015; Wikenheiser and Redish, 2015). The complexity of processes underlying model-based learning exceeds that of basic forms of learning such as Pavlovian conditioning, which can proceed under unaware conditions such as anesthesia or sleep (Weinberger et al., 1984; Fifer et al., 2010). Thus, our position diverges from (Ressler, 2004) who argued that learning *per se* may be used as criterion for consciousness, as illustrated by conditioning in honeybees.

Having identified goal-directed behavior and model-based learning as indicators of consciousness that plausibly apply to primates and rodents, we will briefly compare the evidence with that obtained in another class of animals: birds. Space is lacking to review indicators of consciousness for amphibians, reptiles and invertebrate species, such as cephalopods and insects, noting that detailed knowledge on the indicators of consciousness reviewed here is often lacking for these species. goal-directed behavior and model-based learning have not yet been examined in great depth in non-mammalian tetrapods, but certain bird species display behaviors which are, arguably, tightly linked to goal-directed behavior, such as tool manufacturing and theory of mind (Butler and Cotterill, 2006). For instance, New Caledonian crows (*Corvus moneduloides)* perform complex multi-step procedures in fabricating tools (like wires bent into hooks) to gain access to food, which suggests the presence of goal representations and planning actions forming a causal chain leading up to reaching a pre-set goal (Weir et al., 2002; Butler and Cotterill, 2006; but see Suddendorf and Corballis, 2010). Likewise, theory-of-mind capacities (attributing a mental state to other subjects, allowing to predict the other’s future behavior) have been suggested by specific food re-caching behaviors and deceptive strategies in scrub jays and ravens (Clayton et al., 2003; Bugnyar and Kotrschal, 2004). Because efficacious deceit or misinformation presupposes the representation of a pre-set goal (in this case, to secure cached food in view of competitors able to retrieve the same food), this behavior strongly suggests goal-directed behavior and, *a fortiori*, the internal modeling of another subject’s mental state vis-à-vis one’s own state.

At this point, we have functionally linked consciousness to planned or deliberate goal-directed behavior and model-based learning, but in order to define practically useful indicators of consciousness, it is necessary to scrutinize how tight this linkage may be. If an animal displays goal-directed behavior or model-based learning, does this imply that it *must* be conscious? There are two principal reasons arguing that this relationship is not that straightforward. First, neither the definition of goal-directed behavior nor model-based learning necessarily implies that the subject *must* have a multimodal, situational survey to be able to execute its behavior optimally. For goal-directed behavior, neither the causal action contingency nor the anticipation of outcome value strictly requires consciousness. Similarly, in model-based learning, the acquisition of internal, sensory-specific stimulus-action-outcome models poses no formal requirement on the presence of conscious experience. Internal models of the current world are informative as regards upcoming decisions in two ways: they can be parsed into representations we are aware of (i.e., internally generated models of what is currently going on in our environment to cause the sensations we have) or remain unaware of (models of the causal structure of the environment in terms of what we cannot perceive directly, e.g., unobservable variables that cause you to have a dish with food in front of you).

Second, subjects—whether biological or artificial—may display goal-directed behavior or model-based learning that may arise in a different way than *via* a dependence on conscious experience, no matter how biologically useful this may be. This argument will be developed further in “Consciousness in Intelligent Machines and Its Relations to Animal Cognition” section. In summary, it appears more reasonable to consider goal-directed behavior and related behavioral expressions of model-based learning as indicators of consciousness rather than as indisputable evidence. With “indicator” we mean that a particular type of behavior or neural systems property yields externally observable evidence that the organism under scrutiny is likely to sustain some form of conscious experience—not necessarily having the same phenomenology as human conscious experience, and not necessarily implying proof of consciousness. Given these arguments, as well as other objections to regarding any specific type of non-verbal behavior as sole evidence for consciousness in animals (Weiskrantz, 1995; Seth et al., 2005), it is warranted to widen the search for indicators of consciousness beyond a single indicator of consciousness (such as goal-directed behavior) and next we will consider whether different indicators of consciousness produce a coherent picture for our two case studies (rodents and birds).

### Brain Anatomy and Physiology

The rationale for including these domains of study in our list of indicators of consciousness has been reviewed before and rests on extensive evidence, derived from studies in human patients and non-human primates, that conscious experience is generally dependent on the integrity of thalamocortical systems (Penfield, 1959; Zeki, 1993; Stoerig and Cowey, 1997; Weiskrantz, 1997; Koch, 2004; Key, 2015; Pennartz, 2015). Even though decorticate preparations may still allow interesting and adaptive behaviors to emerge if subcortical systems remain intact (Merker, 2007), extensive evidence on cortical and subcortical brain lesions has linked conscious experience in specific modalities or submodalities to the loss of specific neocortical areas, such as area MT/V5 to conscious motion vision, area V4/V4alpha to color vision, and the ventromedial structures of occipitotemporal cortex (comprising the fusiform and lingual gyri in humans) to form or shape vision (Milner and Goodale, 2008; Karnath et al., 2009; for a discussion of other sensory modalities, see Pennartz, 2015). In the absence of a functioning neocortex, subcortical systems such as the basal ganglia and superior colliculus can continue to mediate sensorimotor behaviors such as orientation responses, feeding behavior and eye movements, but decorticate rats appear to be severely impaired in performing more complex visuospatial behaviors and become easily trapped on platforms and in alleys (Whishaw et al., 1981; for decorticate cats, see Bjursten et al., 1976). Here, we agree with Seth et al. (2005), proposing that animals be considered conscious when their brains possess at least functional analogs of cortex and thalamus.

Even though the rodent thalamocortical system is obviously much smaller in size than that in humans, it contains the same core components, such as the thalamic sensory relay nuclei, the nucleus reticularis, the intralaminar nuclei, and a complex of sensory cortical areas (Krubitzer, 2007) characterized by a hierarchical organization of lower and higher processing stations (Burkhalter, 2016; D’Souza et al., 2016). Similarly, the presence of neuromodulatory systems in the rodent brain stem and mes- and di-encephalon, acting as “enabling factors” for conscious processing, is well recognized, as well as the presence of cortico-basal ganglia thalamic loops subserving selection of action strategies, individual actions, skill learning and long-term goals (Hasselmo, 1995; Groenewegen and Uylings, 2000; Castro-Alamancos and Calcagnotto, 2001; Pennartz et al., 2011). Although major differences between rodent and human brains in terms of size, complexity and the presence of specialized areas should be acknowledged, we argue that the “size” argument will rather affect the complexity and/or intensity of the information the organism will be conscious of, and not so much the presence or absence of consciousness. Furthermore, rodent brains are likely lacking specialized areas such as a well-developed dorsolateral prefrontal cortex, but lesion studies in patients indicate that these areas are not required for consciousness (see e.g., Barbey et al., 2013 on dorsolateral prefrontal cortex). Moreover, prefrontal cortex is present in rodents in at least basic form (Groenewegen and Uylings, 2000; Uylings et al., 2003), and has been implicated in complex cognitive functions (e.g., prospection, evaluation, planning, instrumental and model-based learning, observational learning; Kametani and Kesner, 1989; Kesner et al., 1989; Mulder et al., 2003; Jurado-Parras et al., 2012; Daw and Dayan, 2014; Pezzulo et al., 2014; see also below).

A comparison of brain state physiology between rodents and humans confirms a great cross-species similarity in states associated with wakefulness (conscious state) vs. non-REM sleep (with deep sleep or slow-wave sleep representing an unaware state) and REM sleep (correlated with dream states, regarded as an altered form of consciousness; Hobson, 2009; Pennartz, 2015). Whereas wakeful and REM sleep states are globally characterized in both rodents and primates (including humans) by a desynchronized cortical EEG pattern, by low-power, high-frequency oscillations (e.g., gamma activity) and sparse, irregular firing of thalamic and cortical neurons, deep non-REM sleep is marked by strongly synchronized Delta waves (1–4 Hz), correlating with Down (quiet, hyperpolarized) and Up states (firing, depolarized) in both taxonomic orders. Again, interspecies differences between sleep-wake electrophysiology must be acknowledged, but when comparing rodents with humans, the similarities in the correlations between, on the one hand, wakefulness, REM sleep and desynchronized thalamocortical states, and on the other hand, non-REM sleep and slow-waves, spindles and hippocampal ripples, is striking (e.g., Pennartz et al., 2002; Cantero et al., 2003; Steriade, 2006; Zhang et al., 2018). Thus, the presence of a thalamocortical system displaying similar physiological markers of wakeful and sleep states in rodents is proposed as an indicator of consciousness, although we add that the *absence* of such a system does not imply permanent lack of consciousness, because neural systems may, throughout evolution, have evolved to generate conscious experience in different ways. Moreover, the presence of a thalamocortical system in a desynchronized state may not be *sufficient* for sustaining conscious experience.

As concerns avian brains, it has been noted that a complex of pallial structures shows considerable similarities to the corticothalamic system in mammals, as this complex harbors multiple non-limbic, sensory and associational areas supplied by inputs from many sensory modalities (Butler and Cotterill, 2006). In addition to thalamic relay nuclei and basal ganglia-based loops, avian and mammalian brains share the presence of the thalamic reticular nucleus—as an important structure providing an inhibitory, pacing influence on the palliothalamic or corticothalamic system (Butler and Cotterill, 2006). Although birds lack a claustrum as well as the laminar cytoarchitecture typical of mammalian neocortex, these anatomical features may not be essential for consciousness. Indeed, a comparison of mammalian neocortical structures implied in the perception of different sensory modalities indicates that some “typical” neocortical features are probably not universally required across the modalities, such as a thalamic relay station and a receptive layer 4 in the case of olfaction (Pennartz, 2015). Moreover, the neural dynamics of wakefulness, REM and deep non-REM sleep show the same global patterns of transition in birds as in mammals (Campbell and Tobler, 1984; Kavanau, 2002). It is generally accepted that terrestrial mammals and birds share slow-wave and REM sleep phenomena (Libourel et al., 2018). In conclusion, similarities in brain anatomy and physiology between humans and other vertebrates generally appear to be useful indicators of consciousness and are consistent with some degree of consciousness in rodents and birds. For invertebrates, however, the lack of similarity does not necessarily entail absence of consciousness, necessitating consideration of other criteria.

### Psychometrics and Meta-cognitive Judgment

Following earlier work that established similarities between humans and monkeys in psychometric curves for stimulus detection and discrimination (e.g., Mountcastle et al., 1972; Britten et al., 1992; Spinozzi et al., 2004), more recent studies indicated that also rodents display sigmoid psychometric curves for stimulus detection and discrimination comparable to those of humans (Histed et al., 2012; Brunton et al., 2013; Meijer et al., 2018; see Fay, 1988; Andrew and Greenspan, 1999; Lemon, 2015). While rodents were traditionally viewed as “visually handicapped” due to the lower spatial resolution of their vision (Huberman and Niell, 2011), this is a matter of gradation and should be considered in the context of several visual capabilities at which rodents excel, such as visual contrast sensitivity (Histed et al., 2012; Carandini and Churchland, 2013; Montijn et al., 2015). Moreover, rodents display superb detection and/or discrimination sensitivity in several non-visual modalities (e.g., olfaction, hearing and somatosensation; Fay, 1988; van Duuren et al., 2007; Guo et al., 2014). Their ability to integrate information from multiple senses is close to statistically optimal, as has been found in humans (Raposo et al., 2012). Similarly, detection and discrimination capacities have been well documented in several bird species, and their superior visual acuity and stereovision are particularly striking (Uhlrich et al., 1981; van der Willigen et al., 2010). Taken together with similarities in brain anatomy and physiology (see above), such comparable psychometric performance—found in spite of large differences in brain size and complexity—may be considered an indicator of consciousness, although there is an important caveat that should not be ignored.

Studies on blindsight in humans have made it clear that large lesions of area V1 can preserve detection and discrimination of stimuli that are projected in those parts of the visual field corresponding to the damaged part of the retinotopic map, whereas subjects report that they do not have a visual experience of the presented stimulus. Thus, psychometric performance as tested classically may to some extent be preserved, but when prompted for a “commentary” or meta-cognitive statement about the situation, subjects report an absence of stimulus awareness. In a landmark study, Cowey and Stoerig (1995) showed that monkeys with experimentally controlled V1 lesions exhibit blindsight in that they were able to make above-chance correct discriminations of stimulus location in a forced-choice paradigm, whereas they indicated they had not seen the stimulus when given the option to press a commentary key (which in this paradigm replaced the verbal-response option humans normally have). Although preliminary data from our lab suggest that also rodents can utilize a “Not Seen” response option, caution is warranted for two reasons.

First, even these metacognitive judgment responses can become automatized given a sufficient amount of training or repetition (Weiskrantz, 1995; compare this to a person stereotypically answering “yes, I am listening” to another person who constantly demands attention). This confound is also at play when considering the wider literature on metacognitive judgments in the context of perception, stimulus valuation and consciousness (for examples in the literature on humans, rodents or birds; see Persaud et al., 2007; Kepecs et al., 2008; Seth, 2008; van Wingerden et al., 2012; Watanabe et al., 2014). Thus, caution should be applied when considering potentially habitual expressions of judgment as additional evidence for consciousness. Second, choosing the “Not Seen” option on a physical response panel inevitably coheres with the amount and probability of pay-off attached to this option, compared to other response options. When, for instance, the reward probability for the “Not Seen” option is high compared to one or several of the “Seen” options, the animal may choose “Not Seen” even when it did detect the presented stimulus. To our knowledge, these two problems have not yet been fully resolved in studies on animal behavior, but this can be done in principle by: (i) keeping reward parameters equal for detection vs. no-detection options; and (ii) testing whether responses in these paradigms conform to habitual or goal-directed behavior (see above).

Overall, we propose that the combination of psychometric performance and metacognitive judgment may be used as an indicator of consciousness, if appropriate controls are included to rule out habitual responding or other behavioral confounds.

### Episodic Memory

This type of memory is defined as autobiographical memory, that is memory of events (“what”) a subject experienced at a particular place (“where”) and time (“when”). Together with its decontextualized counterpart, semantic memory, it constitutes declarative memory: memory that humans can consciously recall and verbally report about (Milner et al., 1998; Kandel et al., 2000). Conscious recall is usually understood as being coupled to conscious experiencing of the event before being stored in declarative memory, warranting the proposal that declarative memory and conscious experiencing are tightly and bidirectionally linked to each other (Tulving, 2002; Pennartz, 2018). This opens the possibility to study consciousness through the “backdoor,” i.e., *via* retrievable traces that are left in episodic memory once a consciously experienced event is over. If we can show that certain species display memory capacities similar or identical to those found for human episodic memory, such evidence may provide a strong indicator of consciousness.

Evidence for episodic-like memory in rodents has accumulated over the past decades. Neurons in rodent hippocampus—the brain structure most unambiguously linked to episodic memory—not only display location-coding properties (“place cells”; O’Keefe and Dostrovsky, 1971; Wilson and McNaughton, 1993) but also, depending on behavioral task requirements, a form of temporal coding (“time cells”; Eichenbaum, 2014). The coding of events (“what”), however, is more multi-faceted and greatly depends on the nature of the event and the sensory modalities and submodalities involved. While events such as reward delivery, reward-predicting sensory cues, social interactions or salient but neutral sensory changes in the environment all affect hippocampal coding by firing-rate modulation (Leutgeb et al., 2005; Lansink et al., 2012; Aronov et al., 2017; Danjo et al., 2018), it should be kept in mind that many sensory, spatial and motor-related aspects of consciously experienced events are prominently coded in neocortical areas (see above; e.g., area MT/V5 for motion vision). The causal importance of the hippocampal memory system may not lie in an all-inclusive storage of what-where-when details of the experience, but rather in providing a spatiotemporal scaffold with which individual sensory elements, coded in connected neocortical structures, can be associated during the formation of long-term explicit memory. Upon partial cue presentation, this scaffold can concurrently subserve memory retrieval (pattern completion; Teyler and DiScenna, 1986; Nakazawa et al., 2002; Leutgeb and Leutgeb, 2007; Eichenbaum, 2017).

In addition to neurophysiological research on episodic memory substrates in rodents, behavioral studies have addressed whether rats and mice possess an episodic-like memory that can be behaviorally utilized to retrieve what-where-when knowledge. Some studies suggest that rats are able to remember where, in a radial maze, a preferred reward was previously encountered, and how long ago this happened (Babb and Crystal, 2005; Dere et al., 2006); see also (Veyrac et al., 2015). Other studies, however, suggest that rats can remember some episodic aspects of food encounter events (e.g., location and quality of food items) but lack the capacity to alter caching strategies if food quality is subject to time-dependent decay (Roberts, 2002; Bird et al., 2003; McKenzie et al., 2005; Dere et al., 2006). Thus, whereas electrophysiological recording studies have underscored that the what-where-when aspects of episodic memory are differentially coded in corticohippocampal systems, the integral use of episodic memory in optimizing behavioral strategies remains to be investigated in more detail.

One important aspect of episodic memory should not go unmentioned in this discussion: the capacity for mental time travel (Tulving, 2002). The retrieval of episodic memories enables subjects to generate and re-experience events in the past and future, either in realistic or fictive situations. This has been argued to subserve prospective cognitive processes in both humans and rodents (Corballis, 2013), such as route planning (Pezzulo et al., 2014; Redish, 2016) and internal simulation of behavioral strategies, which can be captured by the term “internal hypothesis testing.” Behavioral and lesioning experiments have yielded evidence for prospective and retrospective cognition in rodents (but see Roberts and Feeney, 2009). In a 12-arm radial maze, Cook et al. (1985) obtained evidence for retrospective and prospective use of spatial memory from the strategies rats deployed to visit food locations in the maze, although, notably, the time span for prospection was less than 1 h. Working in a similar behavioral paradigm addressing potential mental time travel, Kametani and Kesner (1989), Kesner et al. (1989) found that lesions of medial prefrontal cortex and parietal cortex impaired the use of prospective memory strategies.

Extensive work in rodent neurophysiology has raised consistent evidence for the replay of past behavioral experiences during sleep or during behavioral pauses in between action sequences, and this replay of neural sequences generally involves the hippocampus and its connected cortical and subcortical structures (Ji and Wilson, 2007; Pezzulo et al., 2014; Foster, 2017). As argued elsewhere (Pennartz, 2015), replay occurring during hippocampal ripples is probably not coupled to conscious experience. However, the hippocampus has also been shown to generate prospective neural sequences during active locomotion and deliberate (vicarious trial-and-error) behaviors (Johnson and Redish, 2005; Redish, 2016), which is more likely associated with active path planning, goal-directed behavior and wakeful, conscious processing (Kaplan et al., 2017; Pennartz, 2018).

Next to rodents and other mammalian species, evidence for episodic memory in bird species is relatively strong. Clayton and Dickinson (1998), Emery and Clayton (2001), Zentall et al. (2001), Clayton et al. (2003) and Salwiczek et al. (2010) showed that memory in scrub jays is marked not only by “what” happened at a particular location (“where,” i.e., where a food item was cached) but also how much time elapsed since the caching had taken place. In addition, they found evidence for forward planning of actions in the future (e.g., prospective caching) and anticipation of future needs (Correia et al., 2007) which aligns with a capacity for “mental time travel” as another hallmark of episodic memory (Tulving, 2002). However, the latter claim has been criticized by Suddendorf and Corballis (Suddendorf and Corballis, 2010), arguing that food-caching scrub jays did not anticipate on a future desire for specific food, as they did not prefer to store food that would become more desirable in the future. Despite such remaining uncertainties, the case for episodic memory as an indicator of consciousness can be argued to be at least as strong for these bird species as it is for rats or mice.

### Illusion and Multistable Perception

A further indicator of consciousness is related to its definition as multimodal, situational survey, marked by the key feature of intentionality. The subjective nature of conscious experience holds that perceived stimuli or situations may be interpreted in different ways, such as when we view ambiguous stimuli or are subject to illusions. From the perspective of psychophysics, illusion and multistability are important hallmarks of an inferential brain. Basically, it is impossible to experience an illusion unless the brain harbors an internal model that not only accounts for the causes of a particular pattern of sensory input, but also can cause perception to deviate from the actual process generating sensations, in case of ambiguities in their interpretation. Thus, the capacity to misperceive speaks to perception as an active process that discloses an important aspect of conscious processing. As an example of multistable perception, we refer to ambiguous figures such as the Necker cube, where the subject’s percept can switch between two quasi-stable states (e.g., front side of the cube being on the lower right or upper left). Susceptibility to illusions and perceptual ambiguity has been demonstrated in non-human primates and cats (e.g., Logothetis and Schall, 1989; Sheinberg and Logothetis, 1997; Nieder, 2002; Parker et al., 2002; von der Heydt, 2013) and has been utilized to examine whether neurons in lower and higher areas of the visual cortical hierarchy respond to sensory input *per se* or whether their activity correlates with subjective percepts (Leopold and Logothetis, 1999; von der Heydt, 2013; Panagiotaropoulos et al., 2014). Here, behavioral responses to illusion or multistability inducers can be used as an additional indicator of consciousness: specific behavioral patterns can serve to indicate how the subject “acts out” its subjective experience in case it is prone to illusion, whereas a different behavioral response will occur if the subject is not prone to it. Rhesus monkeys, for instance, have been shown to be susceptible to the Rotating Snake illusion (Agrillo et al., 2015; here the inducer of illusory motion is made up of static, interlocking circles that consist of adjacent blue and yellow—or graytone—elements, giving rise to the illusion of seeing the circles rotate, especially in one’s peripheral field of view; Murakami et al., 2006). This susceptibility was expressed by the monkeys making discriminatory choices between static vs. dynamic stimulus arrays. Susceptibility to illusions has been found even in fish, which challenges the hypothesis that cortical substrates are necessarily required to perceive illusory motion (Gori et al., 2014).

As regards rodents, recent evidence suggests that mice are susceptible to the illusion of perceiving large-field contours (Okuyama-Uchimura and Komai, 2016). In a two-choice visual discrimination task, mice were shown to discern illusory rectangular contours based on “Pacman”-type of figures, as often used in the Kanizsa triangle illusion. Further evidence for illusory percepts in mice comes from studies on the rubber tail illusion, which suggests that mice have a sense of body ownership (in this experiment, a mouse’s tail and a fake rubber tail were synchronously stroked, and when subsequently the rubber tail was grasped the mice responded as if their own tail was being touched; Wada et al., 2016). In addition, mice are prone to the motion aftereffect, an illusion of motion that arises after prolonged exposure to motion of an object in one direction (Samonds et al., 2018). Finally, mice have a capacity for amodal completion (Kanizsa et al., 1993), which does not offer evidence for illusion susceptibility *per se* but does argue for an ability to integrate information across large portions of the visual field in order to make inferences about occluded parts of objects. To our knowledge, no evidence has been presented yet in favor of multistable perception in rodents. In conclusion, however, overall the evidence has been accumulating in favor of perception of visual and somatosensory illusions in rodents, and these findings add weight to the overall assessment of consciousness in this taxonomic order.

Similar evidence for susceptibility to illusions and perception of subjective contours has been found in birds. Using a visual setup with grating gaps and phase-shifted abutting gratings, Nieder and Wagner (1999) showed that barn owls can perceive subjective contours that are absent in the retinal image. In single-unit recordings from the visual Wulst area, they identified a significant fraction of neurons generated firing correlates of these contours. In a motion-aftereffect paradigm, Niu et al. (2006) recorded neurons in the pigeon’s pretectum responding to real and subjective contours or producing after-responses to cessation of prolonged motion. Pepperberg and Nakayama (2016) provided evidence for subjective contour perception (Kanizsa figures) as well as amodal completion of occluded figures in the gray parrot. Evidence for multistable perception in pigeons was presented by Vetter et al. (2000), showing that subjects switched in their pecking responses between bistable apparent motion patterns made up by flashing LED displays.

### Visuospatial Behavior

Conscious subjects typically perceive non-mobile objects in their environment as stably positioned, even when they roam their environment and scan it by eye movements. This point is illustrated by monkeys from whom the striate cortex had been surgically removed, resulting in blindsight (Humphrey, 1974; Stoerig and Cowey, 1997; note that other sensory modalities, such as somatosensation, remained intact). Humphrey described a rhesus monkey, Helen, that was nearly without striate cortex and was incapable of detailed shape discrimination or object recognition, but expressed a considerable capacity for visually guided behavior as shown by navigation through a room full of obstacles, by foraging for food items on the floor of a well-known arena, or catching passing flies. Here, the main point of interest in Humphrey’s description lies in two aspects of Helen’s behavior: first, unlike normal monkeys, Helen was unable to reach out to objects that were out of the line of direct sight and had not been foveated just beforehand. Whereas she was able to locate objects in the peripheral visual field by eye movements, she could not utilize visual information from the periphery to guide arm movements. This can be taken as an indication of a lack of visual situational survey.

Even more striking was the observation that the monkey, when confronted with a piece of black tape stuck to the floor in the midst of surrounding obstacles, would try to pick up the object again and again while roaming the arena, failing to notice that this was the same object at the same place, encountered several times before: “every time she moved away and then caught sight of the tape again she appeared to treat it as a new discovery.” Humphrey (1974) concluded that Helen’s visual space was “self-centered,” unable as she was to place objects in a stable spatial framework—which corresponds remarkably well to the feature of consciousness of having a situational survey that has both a dynamic (updatable) and stable nature. Thus, the availability of a stable world representation in the presence of ego-motion can be “acted out” by the subject in its visuospatial behavior, and indeed we propose that this specific type of behavior can be used as yet another indicator of consciousness.

Whether rodents display similar visuospatial behavior, based on constructing a stable world survey, is a question that is hard to answer exactly at present, but it is well known that mice and rats, navigating through an arena, rapidly learn to ignore objects occupying a stable and familiar position in space, whereas they show exploratory behavior (i.e., approaches, sniffing) towards objects with novel properties including a changed location (Bekinschtein et al., 2013; Miranda et al., 2018). It should be noted, however, that the spontaneous location recognition task of Bekinschtein et al. (2013) does not only rely on stable object representation but also on memory for object location. Thus, more rigorous tests of stable object representation within perceived scenes should be designed and tested in rodents.

Notwithstanding the current incompleteness of evidence for visuospatial behavior as an indicator of consciousness in rodents, several other studies provide complementary indications. In a visuospatial attention task, Yang et al. (2017) showed that rats can attend to four different locations on a maze floor in order to conduct stimulus-guided locomotor responses, consistent with an ability to direct attention towards spots in a visuospatial survey. Going back to the decorticate rats studied in Whishaw et al. (1981), it is striking that these animals did not only get easily trapped in relatively simple spatial configurations, but also failed to orient to a location in space where their bodies had been touched just before (Figure 1; an exception being a touch on their snout). When a stimulus was applied to body parts such as shoulders, limbs, paws or tail, the rats could display reactions such as turning or rearing, but their responses were not directed towards the stimulus or to the spatial location where the stimulus had been given. This suggests a failure to integrate spatial information about stimulus location, body positioning and directional responding, consistent with (although not conclusive for) the absence of a multimodal, situational survey in decorticate animals.

Visuospatial behavior has been less intensively investigated in birds than mammals, but it is noteworthy that several bird species show evidence of object constancy in perception. This phenomenon refers to the inference that an object will continue to exist after it has perceptually disappeared and occurs at different levels of complexity. Several Piagetian stages of object constancy have been indicated in ring doves, magpies and African gray parrots, which show behavioral signs of surprise and anger if a moving object that they are tracking disappears and is subsequently replaced by a different, unexpected object (Dumas and Wilkie, 1995; Pepperberg et al., 1997; Butler and Cotterill, 2006). Furthermore, evidence has been gathered to suggest that African gray parrots, facing changes that have been made in a visually presented array of objects, are able to indicate that something in the perceived situation has been changed, as well as what was changed (Pepperberg, 2002).

## Conclusions on Indicators of Consciousness

Which conclusions can be drawn on the validity of the indicators of consciousness for animal consciousness as proposed above? Would satisfaction of only one criterion be sufficient to conclude that a species under scrutiny possesses consciousness—or else, how many indicators of consciousness should minimally be met? First, none of the proposed indicators of consciousness is “hard” in the sense that its satisfaction would offer proof of consciousness. For every indicator of consciousness, it seems possible that one could devise a computational mechanism or multi-area model that could mimic the intended function, without being forced to invoke consciousness in one’s explanation. Yet, this very lack of a one-to-one correspondence between individual computational mechanisms and conscious experience should not be taken to mean that indicators of consciousness are worthless. The basic reason for this gap is that the key features of conscious experience (see “What Does ‘Consciousness’ Mean and What Are Its Key Properties?” section) reflect subjective experience, whereas the proposed indicators of consciousness were set to satisfy the requirement that they be testable by external observation of non-verbal subjects, in this case, animals. Externally observed behaviors, or explanatory schemes or models of neural mechanisms underlying it, should not be expected to reveal directly whether a subject’s experiences are qualitatively rich, have intentionality, etc., because these are not the sort of features that computational models (i.e., models transforming input numbers into output numbers) could be expected to explain or reproduce (Pennartz, 2015). Vice versa, and contrary to Gutfreund (2017), we argue that an increased understanding of the computational mechanisms underlying cognitive phenomena (including conscious experience) in particular animal species does not make it less likely that this species is conscious, because computation and consciousness are not two phenomena standing in opposition to each other: they should be preferably conceived of as forming different levels of representation (computations being situated at a lower representational level than phenomenal consciousness; Pennartz, 2015).

Second, it is notable that for certain taxonomic orders (rodents) or classes (birds) of animals, the assessments across the various indicators of consciousness agree remarkably well with each other, as far as current evidence is permitting. Rodents and birds basically score positively on all indicators of consciousness (goal-directed behavior and model-based learning; anatomy and physiology; psychometrics and metacognitive judgment; episodic memory; illusion induction; visuospatial behavior), although for many criteria more empirical evidence is required to substantiate the particular claims. Rather than emphasizing any singular indicator of consciousness as a criterion for consciousness, we propose that the *consistency* amongst these indicators of consciousness serves to enhance or weaken the case for consciousness in any particular species. This proposal follows the logic that, if there is evidence for *X* AND *Y* AND *Z* (etc., where *X, Y* and *Z* are features of conscious experience), then the probability of some form of consciousness being present is increased. In other words, the integration of scores across the various indicators of consciousness can be used as a *graded* criterion for consciousness, in a similar vein as when unresponsive, brain-damaged patients are subjected to various neurological tests and their scores are summed up to determine the overall grade on the Glasgow Coma Scale (Teasdale et al., 2014). Another comparison coming to mind is nosological psychiatry, having resulted in the Diagnostic and Statistical Manual of Mental Disorders (DSM-5) where a number of symptoms of disorder *X* (e.g., bipolar depression) has to be present for a minimum period of time in order to diagnose the patient as suffering from *X* (American Psychiatric Association, 2013). At the same time, our approach differs from nosological psychiatry because it offers as yet a fairly general heuristic to deal with the problem of consciousness in animals and machines; a concrete and quantitative system (e.g., a linear scale with points) will take more time to develop. We also note that our methodology is species-dependent (e.g., the weight of the anatomy and physiology indicator will be different for mammals vs. invertebrates). Moreover, our indicators refer to larger aggregates of behavioral patterns (e.g., visuospatial behavior) rather than concrete elementary behaviors such as used on the Glasgow Coma Scale.

A logical consequence of a high consistency amongst indicators of consciousness, when applied to certain animal species, is to label the set of behaviors showing this consistency such that this is properly distinguished from behaviors showing either a low consistency or scoring negatively on all points. In analogy to the term “episodic-like memory” that is used for animals that cannot make verbal declarations about what they exactly recall from memory, we propose the term “conscious-like behavior” as a summary term for animals displaying consistently positive scores on behavioral indicators of consciousness. Introducing this term is consistent with abductive reasoning as a heuristic—not, in this case, as a characteristic for how a conscious brain system works, but to assess various consciousness theories relative to one another: one makes an inference to the best explanation of the observable data, pertaining here to animal behavior and other indicators arguing in favor or against consciousness in non-human species.

## Theoretically Derived Measures, Cross-Validation and the Problem of Panpsychism

Consciousness can be decoupled from one’s verbal capacities but can also persist in the absence of observable motor behavior, such as during paralysis, dreaming and locked-in syndrome. It is therefore mandatory to ask whether criteria or indicators of consciousness can be derived from parameters pertaining purely to internal brain processes. Following up on brain physiology (indicator of consciousness #2), EEG recordings can be utilized in clinical practice to derive the bispectral index, which has been proposed as a measure of awareness vs. depth of anesthesia (for a critical review, see Stein and Glick, 2016). Although such indices are useful for evaluating consciousness in humans, they have two significant disadvantages: first, they may be specific to the human brain and may not apply to nervous systems that differ substantially in structure and/or function (e.g., of cephalopods). Second, the range of circumstances under which they are valid is incompletely known as they are not derived from a fundamental theory of consciousness. Indeed, a validated theory of consciousness may, in principle, yield a quantitative measure of consciousness that can be applied in addition to other (e.g., behavioral) criteria. Below we will highlight the importance of cross-validation when trying to apply theoretically derived measures in practice.

To illustrate this, it is instructive to consider Information Integration Theory (IIT; Tononi, 2004; Tononi et al., 2016), which assumes that conscious experience is essentially characterized by both differentiation and integration of information. IIT starts from a conceptual description of consciousness with specific axioms and infers the properties that a physical structure should have to be considered conscious (Oizumi et al., 2014; Tononi et al., 2016). The concept of consciousness IIT refers to is so-called primary consciousness, assumed as being equivalent to experience, which does not require any specific cognitive ability (Massimini and Tononi, 2018). To be conscious, according to IIT, a physical system must function not only through feed-forward mechanisms but also through re-entrant processes: a simple input-output mechanism cannot be conscious because its integrated information (Φ) is zero. Consciousness is equated with maximally integrated information, which is defined as the amount of information generated by a complex of elements, over and above the information of its parts. In turn, information is defined as uncertainty reduction: if more experiential possibilities are ruled out, more information is available and thus Φ increases. In contrast to functionalism, IIT argues that an exclusive focus on functions, ignoring physical structure, cannot explain consciousness (Tononi et al., 2016).

Φ is a gradable measure: different levels of consciousness are possible in different physical systems or even in the same system when this can assume different states of information exchange (e.g., during sleep vs. wakefulness). Even though IIT is not primarily a metaphysical but a scientific theory of consciousness, it defines consciousness as a property that emerges in physical systems organized in a specific way, not as a fundamental feature of reality (Tononi and Koch, 2015). IIT claims that consciousness is identical with the conceptual structure of physical systems characterized by certain postulates, so that consciousness is constitutive and fundamental to such systems, which are not restricted to human brains (Fallon, 2018). For IIT, the states of a neural system that are important for consciousness are those that have maximum cause-effect power on the system itself. Connected neuronal groups have a maximally irreducible cause-effect power on themselves, i.e., they specify a conceptual structure with the highest Φ value. Although in practice Φ cannot be computed for large systems of interconnected elements, it can be indirectly assessed through neuroimaging or electrophysiological measurements (Tononi et al., 2016). When comparing the indicators of consciousness and theoretical framework proposed here with IIT, both frameworks define consciousness by subjective and experiential features. We describe consciousness in representational and inferential terms, conceptualizing consciousness as resulting from inference about the world, from the generative activity of constructing a continuously updated multimodal, situational survey of the world around us and our bodies. In contrast, IIT focuses on information processing in systems of causally connected elements.

Previously some of us have argued (Pennartz, 2015) that IIT’s criterion for ascribing consciousness to a system is insufficient because integrated information processing constitutes only one component of the type of world-modeling activity we call consciousness. Although IIT is useful in thinking about ways to assess consciousness based on internal brain parameters, it is underconstrained and this raises several problems, of which only two can be highlighted here. First, the theory would be forced to attribute some degree of consciousness to too many systems throughout nature, including non-living entities such as weather systems. Weather systems do not qualify for consciousness under different, more conventional criteria. Systems consisting of many elements engaging in causal interactions and showing statistical dependence on one another will result in considerable amounts of Φ, which would move IIT into the domain of panpsychism (Pennartz, 2015). In a blogpost^1^, S. Aaronson reinforced this objection by pointing to mathematical matrices marked by (very) high Φ values, noting that devices such as DVD players are capable of strong information integration based on the n-parity operations they routinely perform. One reply to this objection holds that panpsychism *per se* is not a problem for IIT—on the contrary, it would open up an unconventional view of consciousness that broadens our horizon of things in nature potentially possessing some level of consciousness (Koch, 2012).

A major objection against the argument of panpsychism—as a defense of IIT or in general—relates to a lack of testability, or more specifically, a lack of means for cross-validation. If a given system in nature, e.g., a cyclone, is characterized by a certain amount of Φ, how could one validate that this system is indeed, to some extent, conscious? Do systems that should be considered unmistakeably non-conscious (e.g., DVD players), yet display high Φ values, serve as counterexamples disproving IIT? Vice versa, if a nervous system normally considered to be conscious exhibits low Φ, would this argue against IIT, or does any non-zero Φ value suffice to make the case for consciousness? Thus, a problem for IIT is that no criteria are offered to allow to test whether it is correct or incorrect. On the other hand, one could maintain that our “normal” criteria to label entities as conscious have been simply too narrow. This argument holds that IIT offers a fundamental, new law that does not require cross-validation against more conventional ways of conceptualizing consciousness.

We believe that this position is flawed because, once again, it would make the theory immune against any form of cross-examination and cross-validation. As with all phenomena throughout nature, a particular phenomenon *X* (e.g., magnetism or frozenness) can only be claimed to occur in a system if the system exhibits *manifest* properties indicating that *X* is occurring in that system. For instance, iron but not plastic can be said to have magnetic properties because it displays properties of attraction and repulsion in response to an applied magnetic field, even though both iron and plastic are composed of elementary particles obeying the Standard Model of particle physics, which includes electromagnetic interactions. Similarly, consciousness should be attributed only to systems displaying manifest, observable properties such as signs of wakefulness or specific conscious-like behaviors (see “Indicators of Consciousness in Animals” section) or physiological measures (when validated through their tight correlation with other indicators of consciousness).

Thus, the validity of theoretically derived measures of consciousness can only be assessed by testing them against other, usually externally observable phenomena that researchers can reasonably agree on as being indicative of consciousness. Again, this approach follows the logic of abductive reasoning, which compares hypotheses and infers the better model based on simplicity, effectiveness and the available evidence (Josephson and Josephson, 1996). Typically, we attribute awareness to systems that may also assume unaware states from time to time, such as deep sleep or anesthesia. This attribution follows the logic that the term “unawareness” only finds linguistic use if it arbitrates between contrasting states or conditions (aware vs. unaware). Even when consciousness would be more multifaceted than hitherto acknowledged, the point remains that, if everything in our environment would always be conscious, the concept would lose its usefulness in daily or scientific discourse (Pennartz, 2015).

That IIT is underconstrained as a framework for consciousness raises a second important problem: the quantity it uses to assess consciousness (Φ) does not refer to anything beyond the system’s state it describes. It leaves the key feature of intentionality unresolved, as it does not explain how neural systems can generate contents about something that is different from themselves. In other words, IIT measures do not quantify to what extent a neural (or artificial) system generates beliefs about, or representations of, something. The lack of intentionality in IIT adds to the argumentation related to the problem of panpsychism signaled above.

Which other phenomena, indicative of consciousness, could then be used to test theoretically derived, quantitative measures? Not surprisingly, we refer back to the indicators of consciousness mentioned in “Indicators of Consciousness in Animals” section, noting that they are, on the one hand, linked to the neurorepresentational framework of multimodal, situational survey, but on the other hand are compatible with more than one theory and could thus serve as a more general testbed. Importantly, not only may individual indicators of consciousness provide estimates of conscious state, but especially the consistency amongst scores on different indicators of consciousness can function to provide an overall assessment.

One of the remaining problems is how the conscious status of behaviorally unresponsive patients and other behaviorally incapacitated subjects may be assessed. Currently, no universally accepted criteria are available, but several promising research directions may be indicated. First, decoding fMRI signals evoked by cognitive tasks (e.g., motor imagery) may be used to derive Yes/No answers from unresponsive patients (Laureys et al., 2004; Owen, 2015). A drawback of this approach is that cognition does not entail consciousness; cognitive activity (inferred *via* brain activity correlates) does not necessarily correspond to conscious experience (Jox, 2013; Peterson et al., 2013; Farisco et al., 2015). Second, the complexity of spatiotemporal, cortical EEG patterns evoked by TMS pulses can be used to stratify patients with disorders of consciousness (Gosseries et al., 2014; Casarotto et al., 2016). A third route, derived from the current neurorepresentational theory and currently under development, is to study the differences in representational capacity under aware vs. unaware conditions, i.e., to decode object and scene properties from large amounts of neural signals recorded in parallel and identify those components that are only present during aware but not unaware processing.

All of these neural-processing parameters can be combined with additional measures of local (within-area) and global (across-area) connectivity, such as of coherence, mutual information (and related measures of functional connectivity; e.g., Olcese et al., 2016; Mikulan et al., 2018) and directed information transfer (e.g., transfer entropy; Pal et al., 2016; Olcese et al., 2018a). The practical usefulness of these measures remains to be validated by comparison with clinical assessments either during the period of unresponsiveness or after recovery (Schiff, 2010).

## Consciousness in Intelligent Machines and Its Relations to Animal Cognition

The question of consciousness in intelligent machines, including AI algorithms and, in some cases, mobile robots, will be only briefly discussed here in light of the indicators of consciousness raised here, as it has been the subject of many reviews and books (e.g., Aleksander, 2001; Holland and Goodman, 2003; Reggia, 2013; Pennartz, 2015; Dehaene et al., 2017). The matter has become more urgent with the advent of deep learning neural networks (DLNNs), capable of recognizing complex input patterns and classifying complex scenes (LeCun et al., 2015; Schmidhuber, 2015), but also recent developments in robotics and AI based on deep reinforcement learning call for a reconsideration of the matter. Current reinforcement learning-based networks are capable of supra-human performance on Alpha-Go (Silver et al., 2017) and have been shown to learn 40 computer games simultaneously (Mnih et al., 2015). If intelligent machines would be capable of such advanced processing that they may be considered conscious, it becomes a pressing issue to discuss whether they are entitled to fundamental rights, such as the indemnification of pain and fear, and even the attribution of moral status. It is only 8 years ago that Koch and Tononi (2011) proposed an alternative Turing-type of test for consciousness in machines, namely whether they would be able to answer arbitrary questions about what is going on in a photograph. Today, DLNNs come remarkably close to this, having not only achieved complex scene classification, but also semantic labeling and verbal description of scenes (Karpathy and Fei-Fei, 2015; LeCun et al., 2015).

The fact that consciousness, as we know it, exists in living entities, does not entail that artificial entities could not theoretically be conscious. Several distinctions need to be drawn here. First, with respect to the nature of the “hardware.” In his critique of functionalism as a “scientific deviation as great as that of behaviorism it has attempted to supplant,” Edelman (1992) pointed out that the nature of the software (i.e., consciousness, in his view) perforce depends on the nature of the hardware (here: living brains and central nervous systems, vs. non-living machines), and that understanding the former presupposes knowledge of the latter. Here, the point is that consciousness cannot be understood well without any understanding of that which is conscious. Whereas in traditional computers a clear distinction can be made between hardware and software, it is unclear whether this dichotomy can be similarly applied to the brain and consciousness. Numerous questions arise: if consciousness would exist in an entity which, by its constitutive nature, is materially different from living brains, would it be similar to ours? By what reasoning may we justify an answer? If not, would this affect our abilities to (a) detect its consciousness, (b) understand or gain knowledge of it, and (c) communicate with it, provided some success in (a) and (b)?

Second, should we use the same term “consciousness” to refer to a different kind of entity (e.g., a living body vs. a machine, and thus expand our definition accordingly), or create a new term to denote it? This question is epistemologically very important. On the one hand, linguistic innovations must be well-justified in order to avoid conceptual inflation. Yet, if we are too bound by common usage traditions, we hamper development of new thought. Our languages need to evolve to enable the expression of new ideas, knowledge and normative systems. This is not only a matter of communication but also of thought, for language shapes thought both epistemologically, in terms of what we can think and know, and normatively, in terms of the values we develop. These questions present us with conceptual choices that need to be made both philosophically (in terms of clarity, simplicity and logical coherence) and empirically (in terms of scientific justification, experimental validation and explanatory power).

With these questions in mind, there are multiple reasons to posit that the current generation of DLNNs is not conscious, and *a fortiori*, also has no understanding of presented scenes in a sense that resembles human understanding. Even though the viewpoint that state-of-the-art DLNNs have no consciousness is probably uncontroversial, it is important to expose the reasons why this would be the case. First, even DLNNs that are generally successful in semantically labeling scenes correctly can make rare but gross mistakes which reveal that they entirely miss the gist or emotional significance of a scene, or show that these networks lack any basic understanding of how the world physically “works” (Lake et al., 2017). Even after millions of training trials, current DLNNs are susceptible to erroneous classification that is often based on either irrelevant details of presented scenes, or has no clear correspondence to the way humans reach classification, based in part on our abilities for generalization, conceptual learning and selective attention (Schmidhuber, 2015; Lake et al., 2017). In a similar vein, current AI appears incapable of making counter-intuitive inferences on the basis of basic world knowledge and “common sense” and fails to show the associated ability to manifest artificial stupidity.

This first, empirically derived rationale is related to a second, theoretical argument against DLNN scene understanding and consciousness, which reintroduces the point that conscious experience exists by virtue of the brain’s internally producing a model of one’s current situation—our body and the world currently around us. As state-of-the-art architectures amongst DLNNs, deep convolutional neural networks (DCNNs) are characterized by feed-forward processing of inputs, which are transformed to hidden-layer responses before being converted into output (LeCun et al., 2015). There is no place in this feed-forward scheme to incorporate the genesis of an internal model, in contrast to the key properties laid out in “What Does ‘Consciousness’ Mean and What Are Its Key Properties?” section. For instance, neurons or groups of neurons in a DCNN cannot be claimed to have intentionality or qualitative richness. As currently designed, a DCNN is incapable of solving the basic problem of modality identification, i.e., the problem of representing the sensory modality (e.g., vision) providing inputs to a network as being phenomenally distinct from another modality (e.g., audition). Although several classes of DLNNs are characterized by dense recurrent connectivity, the latter objection applies until proof to the contrary is provided. In this respect, models explicitly aiming to build internal models of the causes of sensory inputs and their environmental settings may offer a more promising avenue for further research (Rao and Ballard, 1999; Bastos et al., 2012; Dora et al., 2018). Recently, generative modeling has found its way into AI, for example in the form of generative adversarial networks (Goodfellow et al., 2014) and variational autoencoders (Kingma and Welling, 2014), offering a potential avenue towards better machine-based scene understanding.

Broadening the discussion to state-of-the-art robots which show “intelligent behavior” in the sense that they can negotiate difficult terrains, solve tasks like opening a door and many other sensorimotor problems (e.g., Murphy et al., 2011), we must next ask whether these artifacts could satisfy one or multiple behavioral indicators of consciousness laid out in “Indicators of Consciousness in Animals” section (Figure 1). For instance, a robot capable of opening a door may be argued to exhibit goal-directed behavior in that it may have been pre-programmed to do so, and has thus, in its software, a representation of a prespecified goal, such as escaping from the building where it was manufactured. Similarly, one may adjust the robot’s software so that it would display the appropriate psychometric responses given sensory stimuli, and program it to generate Seen/Unseen commentary responses in analogy to monkeys with blindsight. It would require a substantial amount of work to endow a robot with behaviorally expressed episodic memory, with appropriate reactions to illusion inducers, and with capacities allowing the robot, while roaming around, to approach and act on objects as being stationary in space (but see Eslami et al., 2018).

Even if these behavioral indicators of consciousness would be met, however, two principal objections remain standing against the claim that such robots are conscious. The first is that intelligent machines may rely on processors which are much faster than neurons, which are limited in speed because of their membrane time constant, axonal and synaptic delays, etc. Neuromorphic chips with acceleration factors in the order of 10^4^–10^5^ as compared to biological neurons have been designed and tested (Friedmann et al., 2013, 2017). In the future, such artificial systems may develop alternative ways to satisfy behavioral indicators of consciousness by computing solutions for goal-directed behavioral problems, episodic memory, etc., in different ways than those evolved through biological evolution. Instead of attempting to generate a multimodal, situational survey of one’s environment and body, a robot may, for instance, have processors adopting a computational strategy of serial accumulation of evidence, based on a one-by-one scanning of elements in the environment, avoiding the need to construct the kind of overall, integrated survey we would normally consider “conscious.” It remains an open question whether this scenario might result in an alternative kind of consciousness. Whether and how alternative, non-conscious strategies would be effective is unknown, but in principle, it appears possible that intelligent machines could display such intelligent, non-automated behaviors without necessarily being conscious.

The second objection relates to the notion that intelligent robots may be pre-programmed to display goal-directed behavior and other conscious-like behaviors. There is nothing in the definition of goal-directed behavior that precludes pre-programmed behaviors from satisfying it. For instance, a humanoid robot such as Honda’s Asimo (Hirose and Ogawa, 2007), on display in Tokyo’s National Museum of Emerging Science and Innovation, is capable of kicking a soccer ball into a goal at a distance—which is a remarkable feat of sensorimotor engineering—but it does so in a pre-programmed fashion, and will stereotypically repeat its action pattern on the next show in the same museum. However, closely related to goal-directed behavior is the concept of model-based learning, which emphasizes the importance of systems that do not have to wait until a command or stimulus arrives in order to produce a desired behavior. Agents trained by model-based learning should be able to improvise in novel or unexpected situations, act on the fly, be flexible and act spontaneously when hitherto familiar circumstances are changing (Daw et al., 2005; Daw and Dayan, 2014; Pezzulo et al., 2014). Thus, a more appropriate test for robots, rather than displaying skilled performance *per se*, is to examine whether they can generalize their previously acquired knowledge to novel situations and produce spontaneous and adaptive improvisations when facing environmental changes that demand complex decisions.

These considerations accumulate to suggest a different approach to machine consciousness than a brief Turing-type of test which only provides a snapshot of a machine’s cognitive capacities obtained by interrogation. This approach should take into account that machines, having much faster processing units at their disposal, may use other strategies for solving complex problems than relying on conscious surveys of the environment (and are thereby less dependent on the functions of consciousness that apply in a biological context), and may display externally observable indicators such as goal-directed behavior by pre-programmed solutions. Thus, the biological function of consciousness proposed above may thus not be simply transferable upon machines, and additional criteria may well be needed. We have reviewed evidence for consciousness in several mammalian and avian species and—although this evidence is not completely unambiguous (and is certainly far from complete)—the force of the combined anatomic, physiological and behavioral-cognitive arguments makes a fairly strong case that rodents and birds do have consciousness. Monkeys make an even stronger case. Here, our point, however, is that also intelligent robots can be best evaluated through a process of prolonged, ethological observation as we have suggested for animals but which may be even more comprehensive in the case of robots in order to exclude pre-programmed solutions. In the case of immobile machines, observation of overt behaviors can be replaced by prolonged tests combining complex sensory stimulation patterns with flexible problem solving, interrogation and analysis of symbolically expressed responses.

The rationale is that the evidence for consciousness will optimally accumulate across the study of multiple behaviors (taxing goal-directed behavior, episodic memory, etc.), which should be maintained under varying environmental circumstances (e.g., with novelty, detours) under which stereotypical, pre-programmed solutions can be excluded. Thus, our proposal to assess machine consciousness is as pluralistic as for animals, but more stringent in excluding AI solutions that need not rely on core features of consciousness. In Antarctic marine habitats, groups of collaborating Orcas display seal hunt behavior in which they collectively generate waves to drive the prey off an ice shelf (see Pitman and Durban, 2012). This is an adaptive, flexible form of behavior arising by virtue of taking into account where an agent’s conspecifics are and what they do, how the ice shelf and sea lion are positioned and respond to oncoming waves, where the animal itself is relative to its fellow hunters and ice shelf, etc.—a complex form of behavior that will typically require a situational survey to produce a catch. If robots will be studied in a similar way, for a prolonged time and probing its reliance on its instantaneous capacity to generate survey updates, a robust test of machine consciousness may be within reach. To the best of our knowledge, no robots have been produced yet that would approach passing such a comprehensive test.

## Author Contributions

CP, MF and KE all contributed to this article. “Biological Function of Consciousness” and “Indicators of Consciousness in Animals” sections were mainly written by CP. “Theoretically Derived Measures, Cross-Validation and the Problem of Panpsychism” section was mainly written by MF and CP. “Consciousness in Intelligent Machines and Its Relations to Animal Cognition” section was mainly written by KE and CP. All authors contributed to “Introduction” section. MF made the tables.

## Conflict of Interest Statement

The authors declare that the research was conducted in the absence of any commercial or financial relationships that could be construed as a potential conflict of interest.

## Footnotes

^1^https://www.scottaaronson.com/blog/?p=1799
